# Supplementary material for: Numerical investigation of sequential phase-locked optical gating of free electrons
Source: Sci Rep. 2023 Nov 2;13:18949. doi: 10.1038/s41598-023-45992-6 (PMC10622506; doi:10.1038/s41598-023-45992-6)
Supplement: Supplementary file 2 — Supplementary Information 1. [file 41598_2023_45992_MOESM2_ESM.docx]

**Sequential phase-locked optical gating of free electrons**

Fatemeh Chahshouri^1*^, Nahid Talebi^1,2,*^

^1^Institute of Experimental and Applied Physics, Kiel University, 24098 Kiel, Germany

^2^Kiel, Nano, Surface, and Interface Science − KiNSIS, Kiel University, 24098 Kiel, Germany

E-Mail: [talebi@physik.uni-kiel.de](mailto:talebi@physik.uni-kiel.de); [Chahshuri@physik.uni-kiel.de](mailto:Chahshuri@physik.uni-kiel.de)

**Supplementary contents:**

Supplementary Note 1: Shaping Electron wave packet with Single Gold Nanorod

Supplementary Note 2: Influence of the Initial Optical Phase on the Electron Energy Spectra

Supplementary Note 3: Elastic Transverse Diffraction of Electron from Dual Interaction System

Supplementary Note 4: Influence of the Wavepacket Dimension

**Supplementary Note 1: Shaping Electron wave packet with Single Gold Nanorod**

To fully understand the impact of the considered gap distance between gold nanorods on their hybridization within the dual interaction system, considering the dynamics of the recoil exerted by a single gold nanorod is important. Figure. S1 demonstrates the electron response to the laser-induced plasmonic near-field of a single nanorod when the initial parameters of the electron and laser in the simulation domain are the same as the dual-interaction scheme. This study resembles the electron modulation occurring within the first near-field zone in the sequential interaction for both direction of laser polarization ($\theta=0^{\circ}$, and $\theta=-30^{\circ}$). Hence, for the small nanorods and short electron wave packet, which are the considered parameters in this work, the gap spacing between these two effectives near-field zone does not lead to the plasmonic hybridization effect^1,2^.


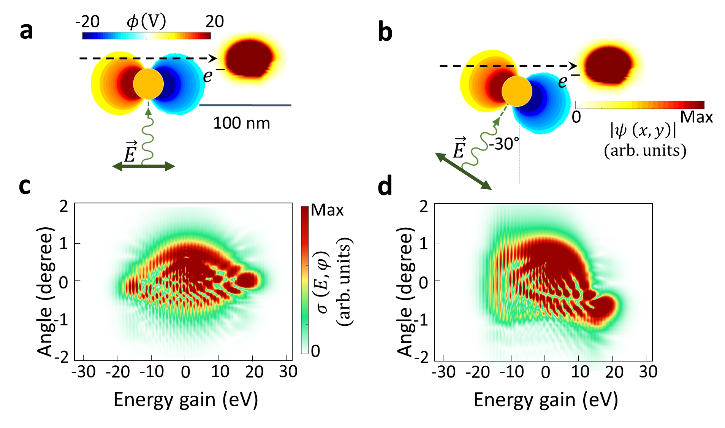


**Figure. S1. Modulation of the electron wavepacket after the interaction with laser-induced dipolar plasmon oscillations in a gold nanorod**. (a) Scalar potential and amplitude of the electron wave function after the interaction with linearly polarized light in (a) normal and (b) oblique excitation scenarios. Inelastic scattering cross-section map corresponding to (c) normal and (d) oblique excitation scenarios, specifying no hybridization in a double interaction system with a 100 nm distance between gold nanorods.

**Supplementary Note 2: Influence of the Initial Optical Phase on the Electron Energy Spectra**


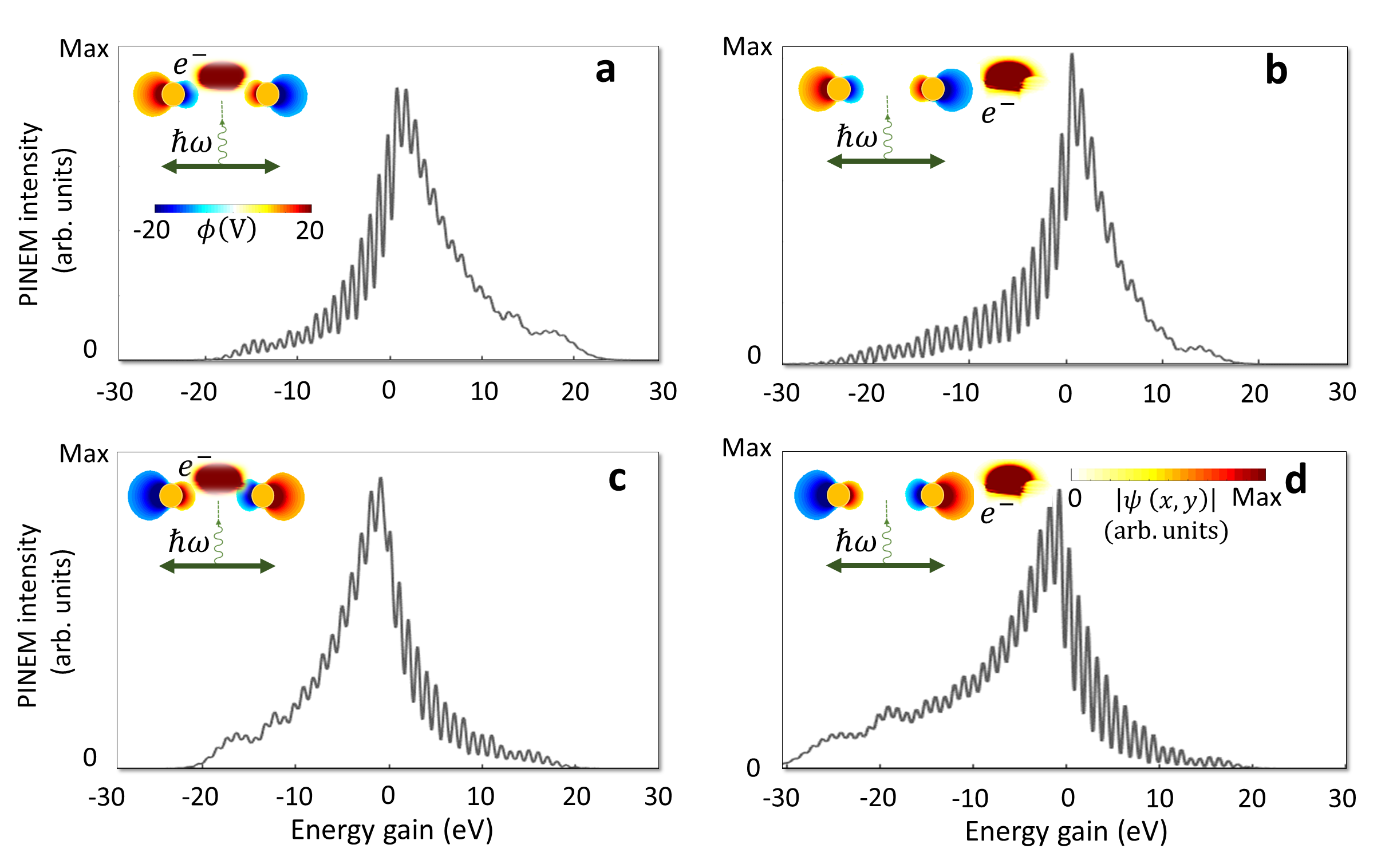


**d**

**Figure. S2. PINEM spectra.** Energy gain spectra after the interaction with the system specified as inset. (a) Single and (b) dual interactions with the depicted positive phase of *x*-polarized initial scaler potential. (c) First and (d) second interactions are initialized by the negative phase of potential. Correlation between the entrance phase and initial interaction mode of the system caused selective accelerated and decelerated PINEM maps.

The final longitudinal energy comb for individual interaction highlights the importance of synchronous motions between near-fields oscillations and electron wavepackets. It has already been shown that in a dual interaction system the combination of the coupling strength from each near-field $g_{1}, g_{2}$ and the phase delay between two spatially varying fields can control the final energy distribution. Here our finding indicates the correlation between the initial phase of the oscillating plasmon fields in final energy modulation of the electron. The PINEM map shows the exchange of $n$ photons with the energies of $\hbar\omega_{ph}$ within the energy range of $-20\mathrm{eV}\leq E\leq20 \mathrm{eV}$. The dual in-phase interaction system for a weak-interaction regime (intensity of zero-line energy peak is more than inelastic peaks) can control the probability amplitude of each photon order. With this the intensity of the spectral peak for already gained electron is doubled, and for the loss channels is suppressed (Figure. S2). When the initial effective phase of the near-field is positive (Figure. S2 a, b) the probability of energy loss events are more than the gain events. This effect is further enhanced in the second interaction zone. The scenario is reversed when the negative lobe of the scaler potential at two spots starts affecting the propagating electron (Figure. S2 c, d).

**Supplementary Note 3: Elastic Transverse Diffraction of Electron from Dual Interaction System**


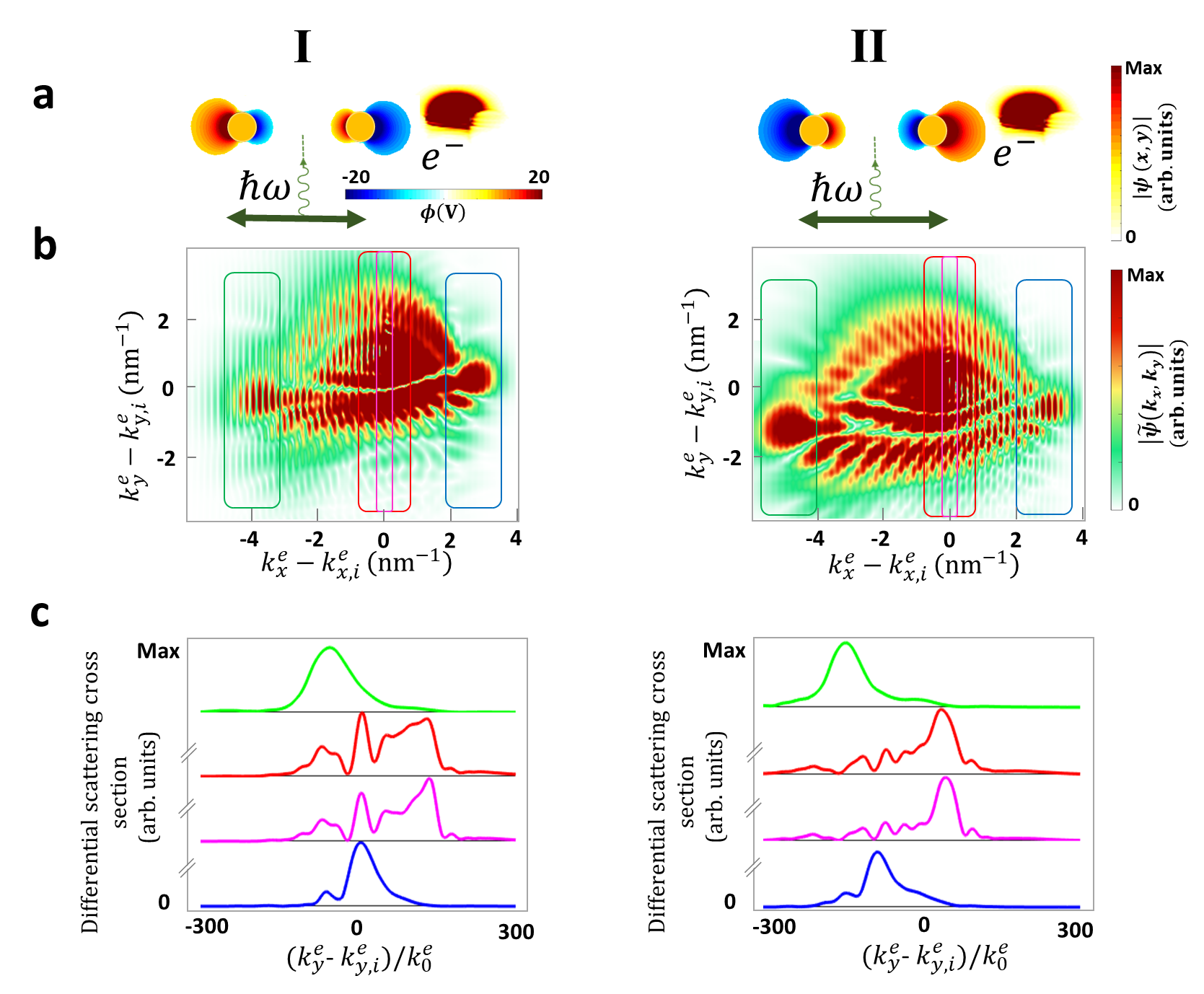


**Figure. S3. Effect of the phase of the near fields on the transverse recoil of free electrons.** The electron initiates its interaction with plasmonic nanorods in a sequential (I) positive, and (II) negative optical phase of the dipolar mode. (a) Schematic of final modulation of the amplitude of the electron wavepacket in real space, and (b) the electron wavepacket in momentum space, after dual interaction with the *x*-polarized dipolar localized plasmons of the gold nanorods. (c) Electron distribution along the transverse direction integrated over the narrow longitudinal ranges shown by the highlighted colored boxes in panel b. The electron has the kinetic energy of 600 eV ($\beta=0.048$), excited with a laser pulse at the wavelength of 700 nm, and field amplitude of $E_{0}=1 \mathrm{GV}m^{-1}$.

To enhance our understanding of the physics of sequential electron interaction with near-field light, we studied the differential scattering cross-section (Figure. S3). To do so, the values of the vertical recoil is averaged over the narrow longitudinal energy ranges (corresponding highlighted energy ranges shown in the inelastic scattering cross section map). Within this configuration, the transversal modulation of the electron wavepacket is better visualized by the line profiles.

The studies for two systems involving subsequent positive (Figure. S3, I), and negative (Figure. S3, II) initial phases of the scaler potential demonstrated the critical role of the arrival time of the electron to the near-fields zone. Assigning the impact of the circular wiggling motion to control the diffraction angle and establish the selection roles for transverse recoil with x-polarized light, we can see distinguishable momentum peaks along positive and negative angles.

As demonstrated in column I of Figure. S3, for electron experience significant elastic scattering (pink, and red boxes), the transversal momentum distribution sustains separate peaks in a diffraction angle close to zero, where the highest intensity after averaging is at zero $k_{y}$. Whereas, for inelastic contributions (green, and blue boxes) there is a sharp diffraction peak broadening close to the zero $k_{y}$. The diffracted peaks in the other system (column II) show two peaks of highly pronounced negative transverse diffraction for inelastically scattered electrons (green, and blue boxes). While for the elastic contribution (pink, and red boxes) it supports one sharp peak at zero and several weaker resonances at negative values of $k_{y}$. Indeed, the relative phase between near-fields in the sequential structure adds another degree of freedom to control the transversal recoil and diffraction peaks and is responsible for producing highly separated spectral fringes.

**Supplementary Note 4: Influence of the Wavepacket Dimension**

Here we provide additional discussion on the effect of the longitudinal broadening of the electron wavepackets on the inelastic energy transfer as well as the elastic diffraction of the electron beam. The simulation parameters here are chosen similar to those presented in Figure. 2a and b of the manuscript with a longer electron pulse. Our simulation shows that involving a larger spatial spread of the electron wavepacket (Figure. S4), which has a longer effective interaction length with the rotational field (4 oscillation period for $80 \mathrm{nm}$, and 6 oscillation period for $120 \mathrm{nm}$), supports the results for the smaller broadening of the electron wave packet. The comparison indicates constructive interference paths originates from the longer electron pulse can ensure discrete and distinguishable momentum modulation like the shorter electron pulses.


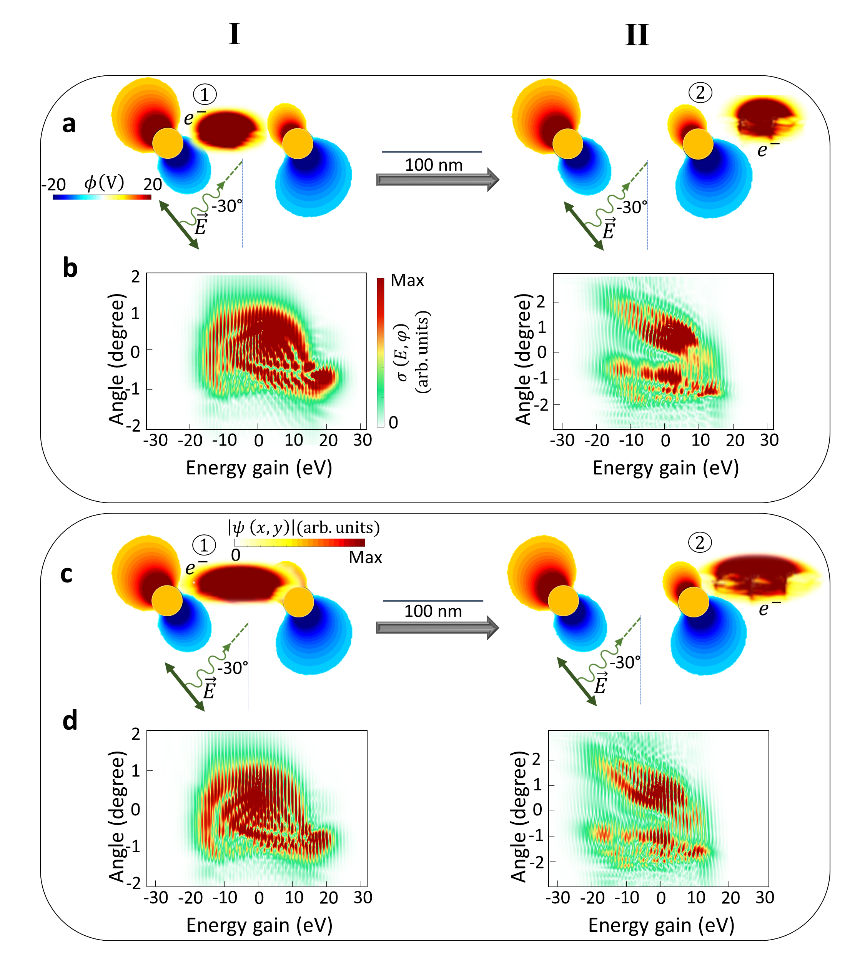


**Figure. S4.** **Effect of the longitudinal broadening of the electron pulse on the final momentum recoil of the electron wavepacket after the sequential interaction.** Final distribution of the transversal and the longitudinal modulation of free electrons in (a, c) real space, and (b, d) its inelastic scattering cross-section after each process (highlighted with 1 and 2 in the figures). The electron wave packet has an initial kinetic energy of 600 eV, with 36 nm transverse broadenings, 80 nm (upper box), and 120 nm (lower box) longitudinal broadenings, respectively (FWHM). Laser electric field amplitude, wavelength, and temporal broadening are $E_{0}=1 \mathrm{GV}m^{-1}$, 700 nm and 18 fs, respectively.

Particularly, for the electron with $120 \mathrm{nm}$ longitudinal broadening, the aforementioned transversal electron deflection in real space is more visible for the bunched electron. This work is experimentally achievable, especially when samples are placed at distances more than $200 \mathrm{nm}$ less than the dispersion length.

**References**

1. Liang, Q. *et al.* Investigating hybridization schemes of coupled split-ring resonators by electron impacts. *Opt Express* **23**, 20721 (2015).

2. Prodan, E., Radloff, C., Halas, N. J. & Nordlander, P. A Hybridization Model for the Plasmon Response of Complex Nanostructures. *Science (1979)* **302**, 419–422 (2003).
